# Effects of dietary *Clostridium butyricum* supplementation on growth performance, intestinal development, and immune response of weaned piglets challenged with lipopolysaccharide

**DOI:** 10.1186/s40104-018-0275-8

**Published:** 2018-08-23

**Authors:** Ling Chen, Shuang Li, Jie Zheng, Wentao Li, Xuemei Jiang, Xilun Zhao, Jian Li, Lianqiang Che, Yan Lin, Shengyu Xu, Bin Feng, Zhengfeng Fang, De Wu

**Affiliations:** 1 Key Laboratory for Animal Disease Resistance Nutrition, Ministry of Education, No. 211, Huimin Road, Wenjiang District, Chengdu, Sichuan 611130 People’s Republic of China; 2 Institute of Animal Nutrition, Sichuan Agricultural University, No. 211, Huimin Road, Wenjiang District, Chengdu, Sichuan 611130 People’s Republic of China

**Keywords:** *Clostridium butyricum*, Growth performance, Immune, Intestinal microflora, Weaned piglets

## Abstract

**Background:**

Weanling pigs, with immature immune system and physiological function, usually experience post-weaning diarrhea. This study determined the effects of dietary *Clostridium butyricum* supplementation on growth performance, diarrhea, and immunity of weaned pigs challenged with lipopolysaccharide (LPS).

**Methods:**

In Experiment (Exp.) 1, 144 weaned piglets were weaned at 21 d and randomly assigned to six groups, with six replicates per group and four pigs per replicate, receiving a control diet (CON) or diet supplemented with antibiotics (AB) or *C. butyricum* (CB) (0.1%, 0.2%, 0.4%, or 0.8%), respectively. All diets in Exp. 1 were a highly digestible basal diet, with 3,000 mg/kg zinc oxide supplied in the first 2 wk only. In Exp. 2, 180 piglets were weaned at 21 d and randomly assigned to five groups, with six replicates per group and six pigs per replicate, receiving CON, AB, or CB (0.2%, 0.4%, or 0.6%) diets. The digestibility of diets was lower than those in Exp. 1, and did not include zinc oxide. At 36 d of Exp. 2, 12 piglets were selected from each of the CON and 0.4% CB groups, six piglets were intraperitoneally injected with LPS (50 μg/kg body weight) and the other six piglets with normal saline; animals were killed at 4 h after injection to collect blood, intestine, and digesta samples for biochemical analysis.

**Results:**

In Exp. 1, CB and AB diets had no effect on growth performance of piglets. In Exp. 2, 0.4% CB decreased feed-gain ratio (*P* < 0.1), diarrhea score (*P* < 0.05), and increased duodenal, jejunal, and ileal villus height and jejunal villus height/crypt depth (*P* < 0.05). The 0.4% CB decreased the plasma tumor necrosis factor (TNF) α (*P* < 0.05) but increased ileal mucosa *IL-10* and *TLR2* mRNA expression (*P* < 0.05). Furthermore, 0.4% CB altered the microbial profile, with *Bacillus* and *Ruminococcaceae UGG-003* at genus level and *Lactobacillus casei* and *Parasutterella secunda* at species level were higher than CON in colonic content (*P* < 0.05).

**Conclusions:**

Dietary *C. butyricum* supplementation had positive effects on growth of weaned piglets with less digestible diets. There was a tendency to reduce the feed-gain ratio, which could reduce feed costs in pig production. Moreover, *C. butyricum* decreased post-weaning diarrhea by improving the intestinal morphology, intestinal microflora profile, and immune function.

## Background

Stress associated with early weaning usually results in depressed feed intake, growth retardation, and post-weaning diarrhea of piglets [1–3]. The sub-therapeutic use of antibiotics as growth promoters has long been recognized as an effective means for the mitigation of weanling stress. Numerous studies have reported that the sub-therapeutic use of antibiotics in diets can promote growth performance and control gastrointestinal infections of weaned piglets [4–6]. However, the ban on use of antibiotics in feed has largely resulted from the emergence of resistant bacteria and the potential for producing drug residues in animal products [7, 8]. Therefore, increasing attention has focused on alternatives to sub-therapeutic antibiotics. The effects of the diet formulation on intestinal development could therefore be critical during the earlier weaning stages.

Direct-fed microbials can improve the growth performance, intestinal health (e.g. intestinal morphology), intestinal microecogical equilibrium, and immunity of piglets [9, 10]. *Clostridium butyricum* can produce butyric acid, and so provide energy for intestinal epithelium and adjust intestinal pH, and maintain the intestinal environment [11]. Previous studies indicated that addition of *C. butyricum* to feed can improve growth performance [12–14], balance intestinal microflora [13], improve intestinal morphology [12], and stimulate the immune system through reducing the expression of pro-inflammatory factors [13, 15, 16]. However, there have been few studies on responses of weaned pigs to *C. butyricum* under lipopolysaccharide (LPS) challenge.

Therefore, this study was conducted to determine the effects of dietary *C. butyricum* supplementation on growth performance, intestinal development, and immune response of weaned piglets with LPS challenge.

## Methods

### Animals and diets

The protocol of this study was approved by the Animal Care and Use Committee of Animal Nutrition Institute, Sichuan Agricultural University, and was carried out in accordance with the National Research Council’s Guide for the Care and Use of Laboratory Animals. In Experiment (Exp.) 1,144 crossbred piglets (Duroc × Landrace × Yorkshire, 7.01 ± 0.03 kg body weight [BW]) were weaned at 21 d of age and randomly assigned to six groups for 28 d, with six replicates per group and four pigs per replicate, receiving a control diet (CON) or diet supplemented with antibiotics (AB) or *C. butyricum* (CB) (0.1%, 0.2%, 0.4%, or 0.8%). All diets were a highly digestible basal diet included highly digestible carbohydrate ingredients (e.g. extruded corn, extruded rice and whey powder) and low anti-nutritional factors protein ingredients (e.g. extruded soybean, spray-dried plasma protein, fishmeal), with 3,000 mg/kg zinc oxide (ZnO) supplied in the first 2 wk only. In Exp. 2, 180 crossbred piglets (Duroc × Landrace × Yorkshire, 6.89 ± 0.02 kg BW) were weaned at 21 d of age and randomly assigned to five groups for 35 d, with six replicates per group and six pigs per replicate, receiving CON, AB, or CB (0.2%, 0.4%, or 0.6%) diets. All diets in Exp. 2 used the same less digestible basal diet without high ZnO. The basal diets of Exp. 2 included lower ratio of high digestibility carbohydrate ingredients and low anti-nutritional factors protein ingredients than Exp. 1, and it did not use rice. Levels of nutrients were provided by the basal diet met the requirements of nutrient requirements of swine (2012). The AB group was supplemented at 1 g/kg diet with (per kg of diet) 75 mg of chlortetracycline and 20 mg of enramycin. The *C. butyricum* strain provided by Chengdu Yukang Technology Co. Ltd. was *Clostridium butyricum* UCN-12, supplemented at 10^8^ CFU/kg. The formulation of basal diets for phase 1 (1–14 d of trial) and 2 (15–28 d) of Exp. 1 are shown in Table 1, and for phase 1 (1–21 d) and 2 (22–35 d) of Exp. 2 in Table 2.

**Table 1:** Composition of basal diet in Exp.1 (% as-fed basis)

Items	Content, %	
	1–14 d	14–28 d
Ingredients		
Corn	19.87	41.53
Extruded corn	19.00	15.00
Extruded rice	10.00	0.00
Extruded soybean	12.00	11.00
Soybean meal	10.00	15.36
Soy oil	0.80	1.58
Spray-dried plasma protein	4.00	0.00
Whey powder	15.00	7.00
Fish meal	5.00	4.00
Sugar	1.50	1.50
*L*-Lysine HCl	0.28	0.44
*DL*-Methionine	0.11	0.09
*L*-Threonine	0.20	0.19
*L*-Tryptophan	0.00	0.01
CaHPO_4_	0.25	0.45
Limestone	0.44	0.60
NaCl	0.15	0.15
Choline chloride	0.10	0.10
Vitamin-mineral premix^a^	1.00	0.00
Vitamin-mineral premix^b^	0.00	1.00
ZnO	0.30	0.00
Total	100.00	100.00
Nutrient composition		
CP	20.56	18.88
DE Mcal/kg	3542	3490
Ca, %	0.8	0.7
Digestible P, %	0.4	0.34
Lys, %	1.35	1.24
Met, %	0.39	0.36
Thr, %	0.79	0.73
Trp, %	0.23	0.2

^a^The premix provided for per kg of feed: Zn, 100 mg; Mn, 4 mg; Fe, 100 mg; Cu, 6 mg; I, 0.14 mg;Se, 0.3 mg; choline chloride, 500 mg; vitamin A, 10,500 IU; vitamin D_3_, 3,300 IU; vitamin E, 22.5 IU; vitamin K_3_, 3 mg; vitamin B_1_, 3 mg; vitamin B_2_, 7.5 mg; vitamin B_6_, 4.5 mg; vitamin B_12_, 0.03 mg; niacin, 30 mg; pantothenate, 15 mg; folic acid, 1.5 mg; biotin, 0.12 mg

^b^The premix provided for per kg of feed: Zn, 80 mg; Mn, 3 mg; Fe,100 mg; Cu, 5 mg; I, 0.14 mg; Se, 0.25 mg; choline chloride, 400 mg vitamin A, 10,500 IU; vitamin D_3_, 3,300 IU; vitamin E, 22.5 IU; vitamin K_3_, 3 mg; vitamin B_1_, 3 mg; vitamin B_2_, 7.5 mg; vitamin B_6_, 4.5 mg; vitamin B_12_, 0.03 mg; niacin, 30 mg; pantothenate, 15 mg; folic acid, 1.5 mg; biotin, 0.12 mg

**Table 2:** Composition of experimental basal diet in Exp.2 (% as-fed basis)

Items	Content, %	
	1–21 d	22–35 d
Ingredients		
Corn	37.35	47.79
Extruded corn	18.00	15.00
Soybean meal	13.00	18.50
Extruded soybean	10.00	6.00
Fish meal	4.00	3.00
Spray-dried plasma protein	3.00	0.00
Whey powder	10.00	5.00
Soy oil	1.03	1.08
CaHPO_4_	0.78	0.66
Limestone	0.95	0.90
NaCl	0.30	0.30
*L*-Lysine HCl	0.32	0.39
*DL*-Methionine	0.16	0.20
*L*-Threonine	0.11	0.16
*L*-Tryptophan	0.00	0.02
Vitamin-mineral premix^a^	1.00	0.00
Vitamin-mineral premix^b^	0.00	1.00
Total	100	100
Nutrient composition		
DE, kcal/kg	3542	3490
CP, %	20.56	18.88
Ca, %	0.8	0.7
Digestible P, %	0.4	0.34
Lys, %	1.35	1.24
Met, %	0.39	0.36
Thr, %	0.79	0.73
Trp, %	0.23	0.20

^a^The premix provided for per kg of feed: Zn, 100 mg; Mn, 4 mg; Fe, 100 mg; Cu, 6 mg; I, 0.14 mg;Se, 0.3 mg; choline chloride, 500 mg; vitamin A, 10,500 IU; vitamin D_3_, 3,300 IU; vitamin E, 22.5 IU; vitamin K_3_, 3 mg; vitamin B_1_, 3 mg; vitamin B_2_, 7.5 mg; vitamin B_6_, 4.5 mg; vitamin B_12_, 0.03 mg; niacin, 30 mg; pantothenate, 15 mg; folic acid, 1.5 mg; biotin, 0.12 mg

^b^The premix provided for per kg of feed: Zn, 80 mg; Mn, 3 mg; Fe,100 mg; Cu, 5 mg; I, 0.14 mg; Se, 0.25 mg; choline chloride, 400 mg; vitamin A, 10,500 IU; vitamin D_3_, 3,300 IU; vitamin E, 22.5 IU; vitamin K_3_, 3 mg; vitamin B_1_, 3 mg; vitamin B_2_, 7.5 mg; vitamin B_6_, 4.5 mg; vitamin B_12_, 0.03 mg; niacin, 30 mg; pantothenate, 15 mg; folic acid, 1.5 mg; biotin, 0.12 mg

Pigs had free access to feed and water. Feed intake and fecal score of each pen was recorded daily. The severity of diarrhea was quantified by using the previous fecal consistency scoring method (fecal scoring: 0, normal; 1, soft feces; 2, mild diarrhea; and 3, severe diarrhea) [17]. Pigs were examined daily to ensure the record, if necessary, therapy of pigs suffering from diseases. Throughout the study, individual piglet BW per pen was measured at 0, 21, and 35 d. In Exp. 2, at 36 d of trial, 12 piglets were selected from each of the CON and 0.4% CB groups, then six piglets were intraperitoneally injected with LPS (50 μg/kg BW) and the othe six piglets with saline. Feed was removed before the injection, and the rectal temperature of each piglet was recorded at 0, 2, and 4 h after injection. The LPS (*Escherichia coli* L2880, Sigma-Aldrich, Los Angeles, CA, USA) was dissolved in sterile saline (9 g/L) to make LPS solution (400 mg/L). Dosage of LPS injection and the time to kill piglets were as previously described [18].

### Sample collection

At 0, 2, and 4 h after injecting LPS or saline, blood samples were collected from the anterior vena cava into heparinized tubes, centrifuged (3,000 r/min at 4 °C for 10 min) and stored at − 20 °C until analysis [19]. The abdominal cavity was opened after being euthanized with an intravenous injection of pentobarbital sodium (50 mg/kg BW). The middle portion (~ 2 cm) of each segment of the small intestine (duodenum, jejunum, and ileum) was sampled and fixed in phosphate-buffered paraformaldehyde for histological measurements as previously described [3]. Ileum segments (10 cm in length) were opened longitudinally and the contents flushed with ice-cold sterile saline. Ileal mucosa and colonic content samples were quickly collected as described previously [20, 21], mucosa was collected by scraping using a sterile glass microscope slide at 4 °C, rapidly frozen in liquid nitrogen and stored at − 80 °C for analysis. Freshly collected contents from the proximal colon were put into sterile Eppendorf tubes and immediately stored at − 80 °C for analyses.

### Intestinal morphology analysis

Intestinal segments were removed from fixative solution and then dehydrated with increasing concentrations of ethanol and chloroform. The segments were processed with paraffin, and two transverse tissue samples were cut from each segment using a microtome. These parts of the tissue samples were dehydrated, embedded together in paraffin wax, and sectioned at 5 μm. One transverse tissue sample of each segment was transferred to a slide and stained with hematoxylin and eosin. Villus height (VH) and crypt depth (CD) were determined as we described previously [3]. Briefly, 10 intact, well-oriented crypt-villi units per sample were randomly selected and measured. The VH was measured from the tip of the villi to the base between individual villi, and CD measurements were taken from the valley between individual villi to the basal membrane.

### Cytokine mRNA abundance analysis

Ileal mucosa samples were used to determine the expression of genes: *TLR2*, *TLR4*, *NF-κB*, tumor necrosis factor α (*TNF-α*), interleukin 6 (*IL-6*), and *IL-10*. Total RNA was extracted from about 50 mg of frozen samples using the RNAiso Plus reagent (TaKaRa Bio, Inc., Dalian, China) according to the manufacturer’s specifications. The RNA concentration in the samples was determined using a DU-800 nucleic and protein detector (Beckman Coulter Inc., Fullerton, CA) at an optical density (OD value) of 260 nm; an OD260:OD280 ratio ranging between 1.8 and 2.0 was considered acceptable. The complementary DNA (cDNA) was then synthesized using a reverse transcription kit (TaKaRa Bio, Inc.) following the manufacturer’s instructions. Primers were synthesized by Invitrogen (Chengdu, China). Real-time PCR was performed on an ABI-7900HT instrument (Bio-Rad, Hercules, CA, USA) to quantify *TLR2*, *TLR4*, *NF-κB*, *TNF-α*, *IL-6* and *IL-10* mRNA expression with a commercial SYBR Green kit (TaKaRa Bio, Inc.). The reference gene β-*actin* was amplified for each sample to verify the presence of cDNA and as an internal control to calculate the relative level of target gene expression using the 2^−ΔΔCT^ method [22]. Relative mRNA expression level of each target gene was normalized to the CON group. Primer sequences are shown in Table 3.

**Table 3:** RT-PCR Primer sequences of target and reference genes

Genes	Primer sequence (5′→3′)	Product, bp	GenBank No.
*TLR2*	F:TCGAAAAGAGCCAGAAAACCAT	58	NM213761
	R:CTTGCACCACTCGCTCTTCA		
*TLR4*	F:AGAAAATATGGCAGAGGTGAAAGC	64	GQ304754
	R:CTTCGTCCTGGCTGGAGTAGA		
*NF-κB*	F:TGCTGGACCCAAGGACATG	60	AK348766.1
	R:CTCCCTTCTGCAACAACACGTA		
*IL-6*	F:GATGCTTCCAATCTGGGTTCA	62	M80258.1
	R:CACAAGACCGGTGGTGATTCT		
*IL-10*	F: GCCTTCGGCCCAGTGAA	71	NM214041.1
	R: AGAGACCCGGTCAGCAACAA		
*TNF-α*	F: TCTATTTTGGGATCATTGCCC	127	NM214022.1
	R: CCAGCCCCTCATTCTCTTTCT		
*β-actin*	F:GGCGCCCAGCACGAT	66	DQ845171.1
	R:CCGATCCACACGGAGTACTTG		

### Plasma pro-inflammatory cytokine concentration analysis

Plasma TNF-α and IL-6 concentrations were measured using the ELISA kits suitable for porcine TNF-α and IL-6 (Nanjing JianCheng Bioengineering Institute Inc.), respectively, according to the manufacture’s protocol. Plasma concentrations of TNF-α and IL-6 were calculated from the standard curve and expressed as ng/L.

### Short chain fatty acid (SCFA) analysis

Colonic SCFAs (acetic, propionic, butyric) were assayed using gas chromatography with a modification of the previous method [3]. Briefly, 1 g of digesta samples was weighed into a 5-mL centrifuge tube and 2 mL of deionized water was added. After the tube was capped, the content vortex-mixed for 30 s, left to stand for 30 min at 4 °C, and then centrifuged (5,000 r/min at 4 °C) for 10 min. The supernatant (1 mL) was removed by aspiration into another 5-mL centrifuge tube, 0.2 mL of 25% metaphosphate and 23.3 μL of 210 mmol/L cortonic acid were added, and this was vortex-mixed for 30 s and left to stand for 30 min. Next, the contents were centrifuged (1,000 r/min at 4 °C) for 10 min. Then 0.3 mL of the supernatant was removed to another 2-mL tube, 0.9 mL carbinol was added, and this was vortex-mixed for 30 s and centrifuged at 1,000 r/min. The supernatant was filtered using a 0.22-μm membrane for gas chromatography analysis.

### 16S rRNA analysis of bacteria

The total genomic DNA of colonic digesta was extracted using a QIAamp DNA stool Mini Kit (Qiagen GmbH, Hilden, Germany) according to the manufacturer’s instructions. Before sequencing, the concentration and purity of the extracted genomic DNA were measured. Integrity of extracted genomic DNA was determined with electrophoresis on a 1% (*w*/*v*) agarose gel. Primer sequencing and bioinformatics analysis were performed by Novogene (Beijing, China) on the Illumina HiSeq platform, using the paired-end sequenced. The V3-V4 region of the bacterial 16S rRNA gene was amplified to comprehensively define the bacterial composition and abundance by PCR using bacterial universal primers. The resulting sequences were clustered into operational taxonomic units (OTUs) using Uparse (Uparse v7.0.1001) at 97% sequence identity. Significant differences were determined through further alpha diversity and beta diversity analyses.

### Statistical analysis

Data were analyzed using SAS (version 9.4; SAS Inst. Inc., Cary, NC, USA). Growth performance of pigs was analyzed using one-way ANOVA to compare the BW, average daily gain (ADG), average daily feed intake (ADFI), feed-gain ratio (F/G), and diarrhea score. The pen was recognized as a statistical unit for the growth performance of pigs. The selected piglet in each pen was taken as an experimental unit for the parameters related to intestinal and immunological function in the LPS challenge study. The parameters related to the inflammatory cytokines in plasma were analyzed by repeated measures analysis with time for the LPS challenge study. For pigs challenged by LPS, data were analyzed using the MIXED procedure, according to the following model:

$$ {Y}_{\mathrm{i}\mathrm{j}\mathrm{k}}=\mu +{\alpha}_{\mathrm{i}}+{\beta}_{\mathrm{j}}+{\gamma}_{\mathrm{k}}+{\left(\alpha \beta \right)}_{\mathrm{i}\mathrm{j}}+{\left(\alpha \gamma \right)}_{\mathrm{i}\mathrm{k}}+{\left(\beta \gamma \right)}_{\mathrm{j}\mathrm{k}}+{\left(\alpha \beta \gamma \right)}_{\mathrm{i}\mathrm{j}\mathrm{k}}+{\upvarepsilon}_{\mathrm{i}\mathrm{j}\mathrm{k},} $$

where *Y*_ijk_ is the analyzed variable, *μ* is the mean, *α*_i_ is the effect of CB (*i* = 1 or 2), *β*_j_ is the effect of LPS (j = 1 or 2), *γ*_k_ is the effect of time (k = 1, 2, or 3), (*αβ*)_ij_ is the interaction between CB and LPS, (*αγ*)_ik_ is the interaction between CB and time, (*βγ*)_jk_ is the interaction between LPS and time, *ε*_ijk_ is the residual error, and (*αβγ*)_ijk_ is the interaction among CB, LPS, and time. Bacteria population data were log-transformed to ensure normal distribution. Values were means with their standard error (SE). Differences were considered significant at *P* < 0.05; when *P* > 0.05 but *P* < 0.1, differences were considered to indicate a trend toward significance. When main effects or interactive effects were significant, the means were compared using the least significant difference method with *P* < 0.05 indicating significance.

## Results

### Growth performance

In Exp. 1, supplementation of CB and AB had no significant effect on growth performance of piglets compared with CON (Table 4). In Exp. 2, 0.4% CB had a tendency to reduce the feed-gain ratio than CON (*P* < 0.1). The 0.4% CB had a lower diarrhea score than CON during the first 3 wk and all period (*P* < 0.05). There were no significant differences in BW, ADG, ADFI, or F/G between the 0.4% CB and AB treatments (Table 5).

**Table 4:** Effect of *C. butyricum* (CB) supplementation on growth performance and diarrhea of weaned pigs in Exp.1^a^

Items	Experimental treatments						Statistics	
	CON	AB	0.1% CB	0.2% CB	0.4% CB	0.8% CB	SEM	*P*-value
BW, kg								
0 d	7.11	7.11	7.10	7.11	7.11	7.11	0.005	0.999
14 d	10.33	10.20	9.87	10.23	10.50	10.27	0.101	0.657
28 d	16.17	16.51	15.44	16.29	16.91	15.40	0.248	0.454
ADFI, g/d								
1–14 d	343.99	334.40	305.91	338.76	349.84	322.17	7.754	0.640
14–28 d	714.72	734.53	652.58	720.94	730.52	641.21	15.851	0.357
1–28 d	529.35	534.47	479.25	529.85	540.18	481.69	10.981	0.398
ADG, g/d								
1–14 d	229.97	220.45	197.83	222.59	248.75	225.51	7.281	0.540
14–28 d	417.26	461.31	397.32	433.04	457.59	367.16	12.731	0.240
1–28 d	323.62	336.83	297.57	327.81	349.97	295.08	8.925	0.443
F/G								
1–14 d	1.50	1.54	1.59	1.53	1.42	1.45	0.023	0.299
14–28 d	1.77	1.60	1.65	1.67	1.60	1.78	0.029	0.291
1–28 d	1.65	1.60	1.63	1.62	1.55	1.66	0.019	0.633
^b^Diarrhea score								
1–14 d	0.17	0.15	0.17	0.15	0.05	0.09	0.019	0.442
14–28 d	0.29	0.18	0.25	0.20	0.21	0.28	0.018	0.439
1–28 d	0.23	0.16	0.21	0.17	0.13	0.19	0.015	0.518

^a^CON, piglets fed the basal diet; AB, piglets fed the basal diet supplemented with 75 mg chlortetracycline and 20 mg enramycin per kilogram; CB, piglets fed the basal diet supplemented with *C. butyricum* preparation;

^b^Diarrhea score = sum of the fecal score / number of test piglets; fecal score: 0, normal; 1, soft feces; 2, mild diarrhea; and 3, severe diarrhea

**Table 5:** Effect of *C. butyricum* (CB) supplementation on growth performance and diarrhea of weaned pigs in Exp.2^c^

Items	Experimental treatments					SEM	*P*-value
	CON	AB	0.2% CB	0.4% CB	0.6% CB		
BW, kg							
0 d	6.90	6.90	6.90	6.90	6.90	0.000	0.772
21 d	8.54	9.17	8.63	8.70	8.64	0.087	0.147
35 d	14.72	15.60	14.75	14.80	14.46	0.141	0.105
ADFI, g/d							
1–21 d	192.82	205.56	188.4	188.83	185.27	3.450	0.392
21–35 d	696.41	731.27	725.22	696.01	687.34	9.756	0.546
1–35 d	394.26	415.85	403.17	391.71	386.10	5.168	0.416
ADG, g/d							
1–21 d	119.14	145.99	118.83	126.77	124.22	3.635	0.100
21–35 d	458.97	459.25	437.65	436.07	437.41	6.139	0.567
1–35 d	223.52	248.70	224.52	225.89	217.23	3.945	0.103
F/G							
1–21 d	1.65^a^	1.42^b^	1.59^a^	1.49^ab^	1.50^ab^	0.031	0.033
21–35 d	1.52	1.59	1.66	1.60	1.58	0.019	0.243
1–35 d	1.77	1.68	1.80	1.74	1.78	0.015	0.099
^d^Diarrhea score							
1–21 d	0.97^a^	0.86^b^	0.86^b^	0.79^b^	0.81^b^	0.018	0.007
21–35 d	0.13	0.10	0.08	0.06	0.12	0.012	0.306
1–35 d	0.64^a^	0.55^b^	0.55^b^	0.50^b^	0.54^b^	0.013	0.003

^a, b^Means in the same row with different superscript letters differ significantly (*P* < 0.05)

^c^CON, piglets fed the basal diet; AB, piglets fed the basal diet supplemented with 75 mg chlortetracycline and 20 mg enramycin per kilogram; CB, piglets fed the basal diet supplemented with *C. butyricum* preparation;

^d^Diarrhea score = sum of the fecal score / number of test piglets; fecal score: 0, normal; 1, soft feces; 2, mild diarrhea; and 3, severe diarrhea

### Changes of rectal temperature

**Fig. 1:**
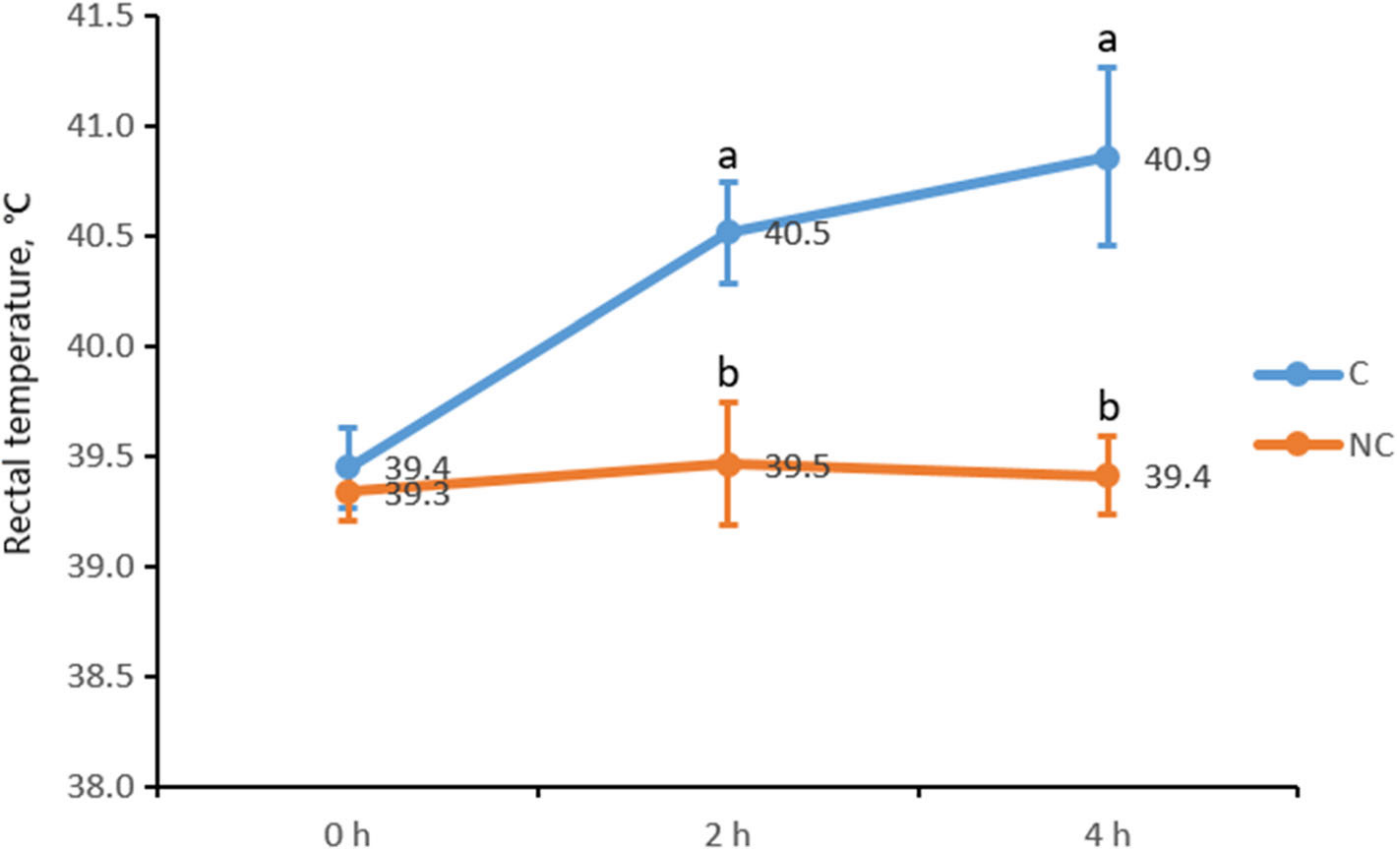
Changes of rectal temperature. Weaned piglets challenged with LPS (C) and not challenged with LPS (NC). ^a, b^ Mean values with unlike letters were significantly different (*P* < 0.05)

Compared with the non-challenged piglets, the rectal temperature of LPS-challenged piglets increased significantly at 2 and 4 h (Fig. 1).

### Intestinal morphology

The 0.4% CB significantly increased duodenal, jejunal and ileal VH and jejunal VH/CD (*P* < 0.05); whereas VH, CD and VH/CD were not affected by LPS or the CB × LPS interaction. Duodenal VH and VH/CD in the CB + LPS were higher (*P* < 0.05) than those in the CON + LPS; jejunal VH and VH/CD were higher (*P* < 0.05) in the CB – LPS than in the CON – LPS; and ileal VH was higher (*P* < 0.05) in the CB + LPS than in the CON + LPS (Table 6).

**Table 6:** Effect of *C. butyricum* (CB) supplementation on intestinal morphology of weaned pigs challenged with LPS^d^

Items		–LPS		+LPS		SEM	*P*-value		
		CON	CB	CON	CB		CB	LPS	CB × LPS
Duodenum	VH, μm	223.89^b^	277.70^b^	241.57^b^	340.81^a^	10.38	0.001	0.066	0.287
	CD, μm	241.39	279.93	314.23	288.52	14.64	0.829	0.180	0.286
	VH/CD	0.94^ab^	1.02^ab^	0.85^b^	1.25^a^	0.06	0.060	0.575	0.202
Jejunum	VH, μm	201.46^c^	277.39^a^	226.91^bc^	251.38^ab^	7.40	0.003	0.985	0.097
	CD, μm	171.26	171.24	198.06	174.73	7.61	0.452	0.331	0.453
	VH/CD	1.20^b^	1.69^a^	1.19^b^	1.48^ab^	0.07	0.010	0.442	0.470
Ileum	VH, μm	211.19^ab^	220.89^ab^	179.60^b^	248.97^a^	8.68	0.034	0.920	0.101
	CD, μm	160.27	188.33	189.47	163.31	9.26	0.959	0.911	0.159
	VH/CD	1.40	1.60	0.96	1.60	0.10	0.185	0.692	0.085

^a, b, c^Means in the same row with different superscript letters differ significantly (*P* < 0.05)

^d^CON, piglets fed the basal diet; CB, piglets fed the basal diet supplemented with 0.4% *C. butyricum* preparation; −LPS, piglets not challenged with LPS; +LPS, piglets challenged with LPS

### Plasma TNF-α and IL-6 concentrations, and ileum mRNA expression

Plasma TNF-α concentration was affected by LPS challenge (*P* < 0.1) and CB × LPS interaction (*P* < 0.05); plasma IL-6 concentration was affected by LPS (*P* < 0.1). Plasma TNF-α concentration averaged across time was higher (*P* < 0.05) in the CON + LPS than the CON – LPS treatment, but no difference was observed between the CB – LPS and CB + LPS treatments (Table 7).

**Table 7:** Effect of *C. butyricum* (CB) supplementation on plasma cytokine concentrations of weaned pigs challenged with LPS^c^

Items		–LPS		+LPS		SEM	*P*-value						
		CON	CB	CON	CB		CB	LPS	Time	CB × LPS	CB × Time	LPS × Time	CB × LPS × Time
IL-6, ng/L	0 h	222.99	204.67	220.53	208.16	3.511	0.235	0.064	0.268	0.303	0.391	0.418	0.480
	2 h	209.61	200.82	239.33	216.21								
	4 h	207.11	230.70	243.49	230.91								
	Average	213.24	212.06	234.45	218.43								
TNF-α, ng/L	0 h	211.27	245.28	253.01	239.81	4.384	0.471	0.063	0.213	0.038	0.308	0.973	0.94
	2 h	231.11	240.78	268.07	242.16								
	4 h	250.86	246.20	282.36	243.80								
	Average	231.08^b^	244.09^ab^	267.812^a^	241.92^ab^								

^a, b^Means in the same row with different superscript letters differ significantly (*P* < 0.05)

^c^CON, piglets fed the basal diet; CB, piglets fed the basal diet supplemented with 0.4% *C. butyricum* preparation; −LPS, piglets not challenged with LPS; +LPS, piglets challenged with LPS

**Fig. 2:**
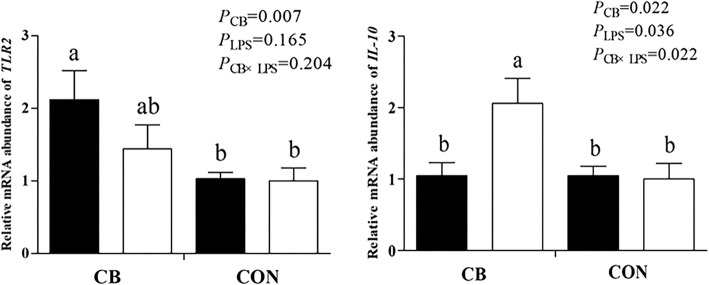
Relative mRNA expression *TLR2* and *IL-10* of ileal mucosa. Weaned piglets challenged with LPS(■) and not challenged with LPS(□). Values are means with their standard errors represented by vertical bars. ^a, b^ Mean values with unlike letters were significantly different (*P* < 0.05)

The 0.4% CB increased ileum mRNA relative expression of *TLR2* and *IL-10* (*P* < 0.05). The LPS decreased the mRNA relative expression of *IL-10* (*P* < 0.05), and there was a significant CB × LPS interaction (*P* < 0.05) (Fig. 2).

### SCFA concentrations

The acetate concentration in colonic content was affected by LPS challenge (*P* < 0.1). Concentrations of acetate, propionic acid, and butyric acid in colonic content were not affected by CB and CB × LPS (*P* > 0.05) (Table 8).

**Table 8:** Effect of *C. butyricum* (CB) supplementation on SCFA concentrations in colonic and cecal content of weaned pigs challenged with LPS^a^

Items	–LPS		+LPS		SEM	*P*-value		
	CON	CB	CON	CB		CB	LPS	CB × LPS
Colonic content, μmol/g								
Acetate	35.58	33.27	30.24	29.14	1.357	0.537	0.096	0.824
Propionic acid	20.28	16.38	16.97	16.41	0.808	0.182	0.322	0.313
Butyric acid	7.09	7.79	6.80	5.83	0.419	0.874	0.195	0.334

^a^CON, piglets fed the basal diet; CB, piglets fed the basal diet supplemented with 0.4% *C. butyricum* preparation; −LPS, piglets not challenged with LPS; +LPS, piglets challenged with LPS

### Microbial community in colonic content

#### DNA sequence data and OTU clustering

**Fig. 3:**
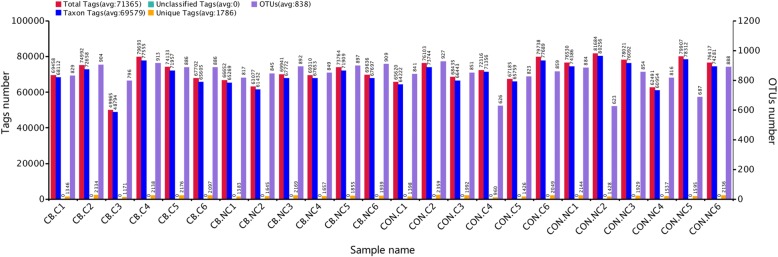
OTUs clustering and annotation per sample. Piglets in CB treatment challenged with LPS (CB.C) and not challenged with LPS (CB.NC). Piglets in CON treatment challenged with LPS (CON.C) and not challenged with LPS (CON.NC)

A total of 1,712,770 effective tags were obtained from four groups, with an average of 71,365 ± 1,437 per sample. Further study of the species diversity of the samples and species annotated on the representative sequence of OTUs. A total of 20,102 OTUs were found in the four groups, with an average of 838 ± 17 per sample (Fig. 3).

#### Alpha diversity of microbial community in colonic content

Many indexes that represented alpha diversity of microbial community (Table 9), in addition to the observed-species and ACE, were higher in the CB than in the CON (*P* < 0.1), indicating significantly greater species richness.

**Table 9:** Effect of *C. butyricum* (CB) supplementation on alpha diversity of microbial community in colonic content of piglets challenged with LPS^a^

Items	–LPS		+LPS		SEM	*P*-value		
	CON	CB	CON	CB		CB	CON	CB × LPS
Observed-species	731.67	801.00	751.17	801.50	15.849	0.074	0.756	0.767
Shannon	6.888	7.145	6.828	7.134	0.125	0.274	0.889	0.924
Simpson	0.976	0.980	0.966	0.980	0.004	0.307	0.519	0.556
Chao1	822.53	853.28	803.84	857.06	16.771	0.225	0.826	0.741
ACE	809.26	868.06	809.00	865.86	16.099	0.088	0.97	0.976
Goods-coverage	0.998	0.998	0.988	0.998	0.000	0.329	0.329	0.329

^a^CON, piglets fed the basal diet; CB, piglets fed the basal diet supplemented with 0.4% *C. butyricum* preparation; −LPS, piglets not challenged with LPS; +LPS, piglets challenged with LPS

#### Change of relative abundance at phylum and genus levels

**Fig. 4:**
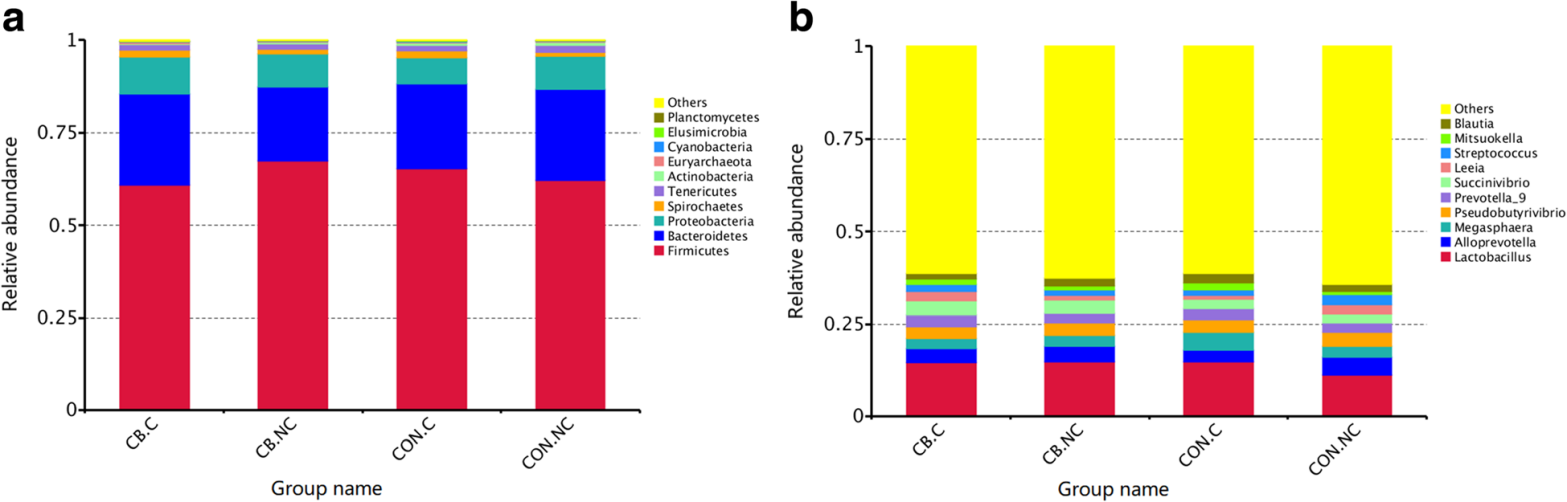
The relative abundance of microbiota at phylum (**a**) and genus (**b**) level. Piglets in CB treatment challenged with LPS (CB.C) and not challenged with LPS (CB.NC). Piglets in CON treatment challenged with LPS (CON.C) and not challenged with LPS (CON.NC)

A total of 24 phyla were shared by piglets from all groups, and seven bacteria had relative abundance exceeding 1% in at least one sample: Firmicutes, Bacteroidetes, Proteobacteria, Spirochaetes, Tenericutes, Actinobacteria, and Euryarchaeota. The top 10 phyla are shown in Fig. 4a and relative abundances of the top 10 genera are show in Fig. 4b. There were no significant differences for the top 10 at phylum and genus levels among all groups.

#### Analysis of different species among groups

**Fig. 5:**
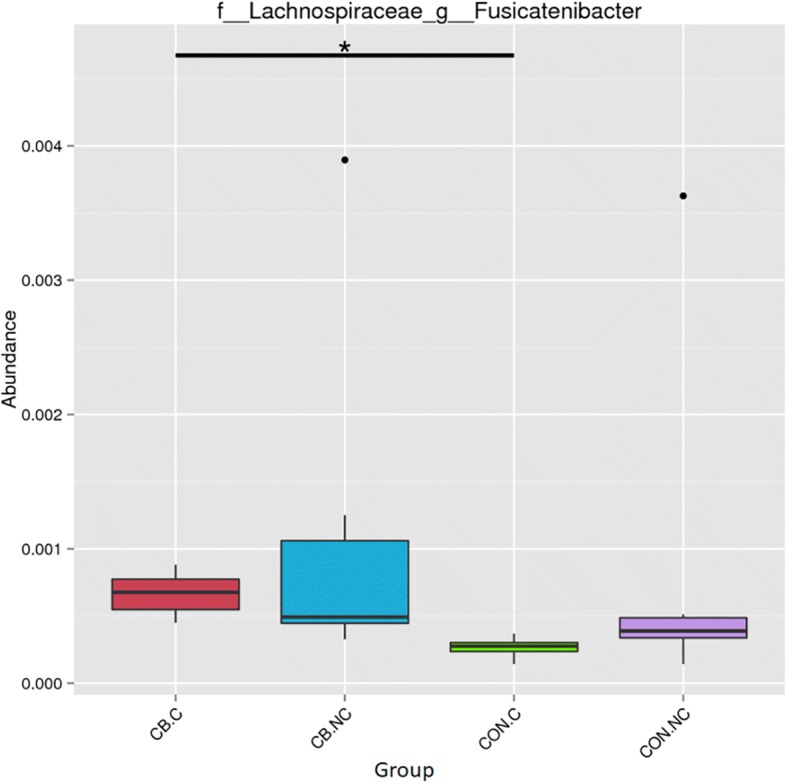
The box graph of significant differences among species. The cross line represents two groups with significant differences, and no cross line indicates that there is no difference between the two groups. “*” indicates significant differences between the two groups. Piglets in CB treatment challenged with LPS (CB.C) and not challenged with LPS (CB.NC). Piglets in CON treatment challenged with LPS (CON.C) and not challenged with LPS (CON.NC)

**Fig. 6:**
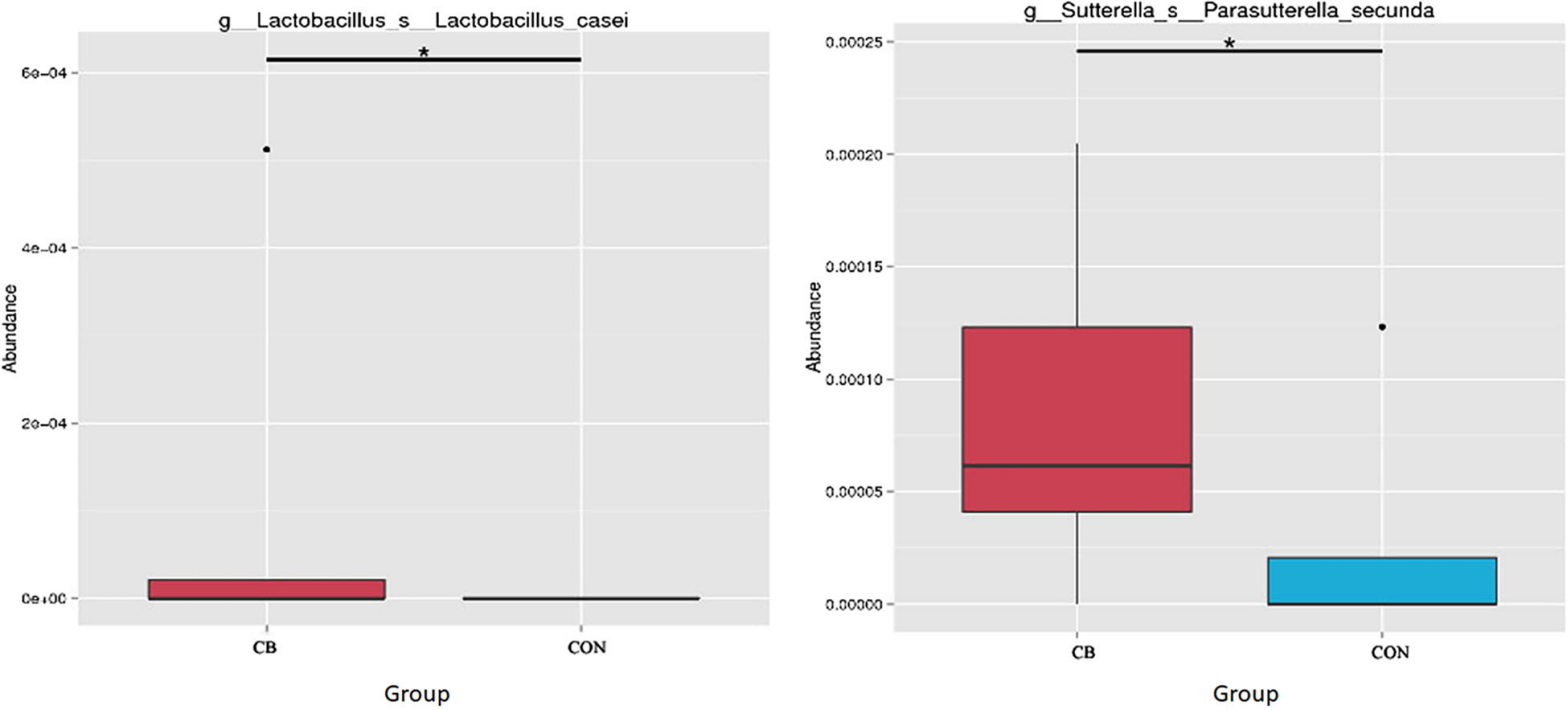
The abundance of species at species level. The cross line represents two groups with significant differences, and no cross line indicates that there is no difference between the two groups. “*” indicates significant differences between the two groups. Piglets in CB treatment challenged with LPS (CB.C) and not challenged with LPS (CB.NC). Piglets in CON treatment challenged with LPS (CON.C) and not challenged with LPS (CON.NC)

**Fig. 7:**
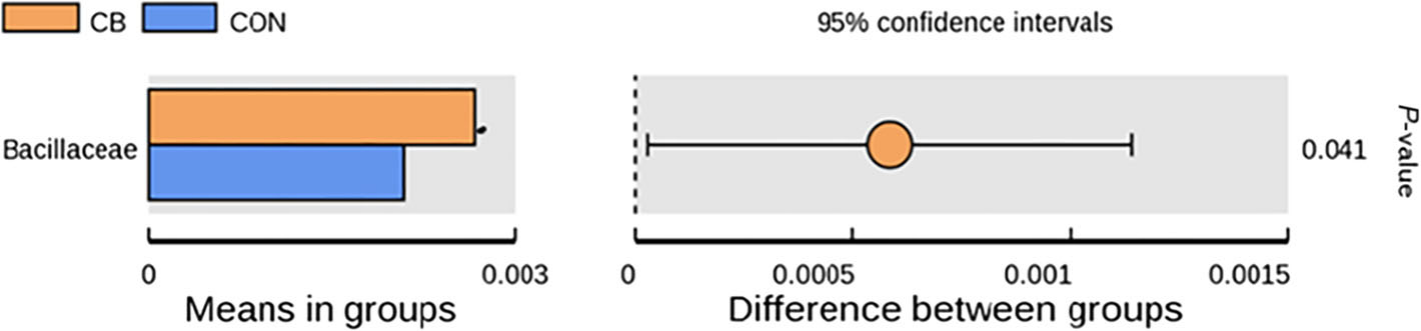
The species of significant differences at family level. The left picture shows the diversity of species abundance, each of which indicates the mean value of species with significant differences in the abundance between groups. The right picture shows the difference confidence between groups. The most left-hand point of each circle represents the lower limit of the 95% confidence interval of mean difference, and the most right end point of the circle represents the upper limit of mean difference and 95% confidence interval. The center of the circle represents the difference of the mean. The group represented by the circle color is a group with high mean value. The right end of the display results was the *P-*value of significance test for the corresponding species between groups. Piglets in CB treatment challenged with LPS (CB.C) and not challenged with LPS (CB.NC). Piglets in CON treatment challenged with LPS (CON.C) and not challenged with LPS (CON.NC)

**Fig. 8:**
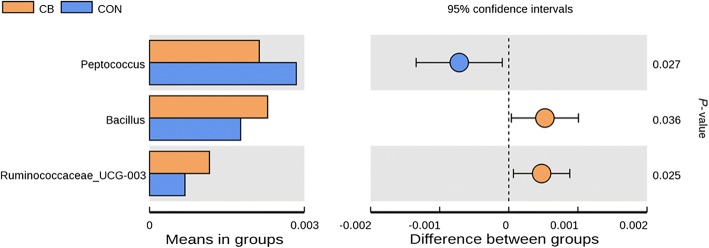
The species of significant differences at genus level. The left picture shows the diversity of species abundance, each of which indicates the mean value of species with significant differences in the abundance between groups. The right picture shows the difference confidence between groups. The most left-hand point of each circle represents the lower limit of the 95% confidence interval of mean difference, and the most right end point of the circle represents the upper limit of mean difference and 95% confidence interval. The center of the circle represents the difference of the mean. The group represented by the circle color is a group with high mean value. The right end of the display results was the *P-*value of significance test for the corresponding species between groups. Piglets in CB treatment challenged with LPS (CB.C) and not challenged with LPS (CB.NC). Piglets in CON treatment challenged with LPS (CON.C) and not challenged with LPS (CON.NC)

The abundance of *Fusicatenibacter* at genus level was higher in the CB.C than in the CON.C (Fig. 5). The abundances of *Lactobacillus casei* and *Parasutterella secunda* at species level were higher in the CB than CON (Fig. 6). The t-test show that greater abundance of Bacillaceae at family level in the CB than the CON (Fig. 7) and abundances of *Bacillus* and *Ruminococcaceae UGG-003* at genus level were higher in the CB than the CON; however, abundance of *Peptococcus* at genus level was lower in the CB than the CON (Fig. 8).

## Discussion

Many reports have shown that *C. butyricum* can promote growth performance and improve nutrient utilization [13, 14, 16, 23], but other studies have no effect on growth performance [24]. Consistent with previous studies, dietary *C. butyricum* supplementation decreased diarrhea score in Exp. 2, but the supplementation had no effect on growth performance and diarrhea score in Exp. 1. This discrepancy might be related to diet type, such as the different percentages of highly digestible ingredients between the Exps 1 and 2. In the diet formulation of Exp. 1, we attempted to maximize the inclusion of various highly digestible carbohydrate ingredients and reduce anti-nutritional factors. This was because high quality protein sources and a high digestibility of carbohydrate sources were necessary for weaned pigs, to avoid the negative effects associated with post-weaning performance. Previous work suggested that ZnO and antibiotics are beneficial to growth and decrease diarrhea [6, 25]. The purpose of using ZnO was to prevent severe diarrhea of piglets in Exp. 1, and resulted in no significant severe diarrhea among groups; the growth performance did not differ significantly between AB and CB groups. The *C. butyricum* reduced diarrhea for low digestibility diets without antibiotics and ZnO in Exp. 2, which would be very beneficial to reduce costs in commercial production. This study showed that 0.4% CB improved feed efficiency and decreased diarrhea score compared with CON, and with no significant difference to AB, showing that *C. butyricum* had positive effects and similar growth-promoting effects to antibiotics with the less digestible diet. Previous studies found that dietary supplementation with direct-fed microbials could reduce the frequency of post-weaning diarrhea in piglets, reduce diarrhea severity, and provide greater growth rate and feed efficiency [26]. Oral administration of *C. butyricum* as a direct-fed microbial is gaining importance in treating and improving animal performance [15].

Intestinal histomorphology had been widely used for assessing intestinal development and function [27, 28]. The decreased digestion and absorption of nutrients due to villous atrophy and crypt hypertrophy as a result of early weaning may contribute to diarrhea [29, 30]. The underlying mechanism is related to the fact that increased VH and VH/CD are directly correlated with increased epithelial turnover [31], and longer villi are linked with activation of cell mitosis, with shortening of villi and deeper crypts leading to poor nutrient absorption [32], increased secretion in the gastrointestinal tract and reduced performance [33]. Previous studies indicated that direct-fed microbials could promote intestinal development and so improve piglet health and the growth performance [9, 10, 34, 35]. Consistent with this, some studies reported that use of *C. butyricum* in diets for weaned piglets could improve weight gain and feed efficiency when used at an appropriate dose [36]. In our study, supplementation of *C. butyricum* in the diet of weaned piglets consistently increased the VH of duodenum and ileum [37], and the VH/CD significantly increased [29, 38], which indicated the better digestive and absorption capability and resulted in the decreased F/G [34].

The lower diarrhea score, for piglets receiving *C. butyricum*, suggested a healthier gastrointestinal environment, possibly associated with intestine development, simultaneously, changes in intestinal microbiota and immunity were also possible. Previous studies indicated that direct-fed microbials in diets can significantly improve immune response [39, 40]. In line with this, dietary supplementation with *C. butyricum* has promoted immune response and improved intestinal barrier function in broiler chickens, rats and ducks [16, 31, 41]. The present study, the increased body temperature and plasma TNFα and IL-6 concentrations indicated successful establishment of the immune model following LPS challenge. The current results showed that the inflammatory process might be modulated by *C. butyricum*, as shown by results indicating decreased pro-inflammatory cytokine TNF-α and increased *IL-10* and *TLR2* expressions. The molecular action mechanism of *C. butyricum* involved reduced inflammation and improved immune homeostasis [42]. Mucosal surfaces of the gastrointestinal tract are in continuous contact with microbes, and toll-like receptors (TLRs) mediate recognition of microbial molecules to generate immune response [43]. The *C. butyricum* was shown to drive secretion of MyD88-independent inflammatory cytokines via TLR2-induced NF-κB activation [15], and *C. butyricum* can induce *IL-10* expression from intestinal macrophages through the TLR2/MyD88-mediated pathway [44] consistent with our results showing *C. butyricum* increasing *TLR2* and *IL-10* expressions. The IL-10 is one of the most potent anti-inflammatory cytokines and is required for protection in many animal models of inflammation, and it has important roles in the regulation of gut homeostasis during host defense [45, 46]. The association between IL-10 and inflammatory bowel disease has been demonstrated in both humans and in animal models [46].

The TLR/MyD88-signal pathway triggers several responses critical for maintaining host-microbial homesostasis [47]. Opportunistic invasion of host tissue by resident bacteria has serious health consequences including inflammation and sepsis. The immune system has thus evolved adaptations that work together to contain the microbiota and preserve the host-microbiota symbiotic relationship [47]. The intestinal tract harbors a complex microbial community that plays a key role in nutrition and health, and the colon is the main site of microbial colonization [45]. Failure to achieve or maintain equilibrium between a host and its microbiota has negative consequences for both intestinal and systemic health, likely resulting not only in intestinal inflammatory diseases [40], such as Crohn’s disease and ulcerative colitis, but might also contribute to “auto-immune” diseases at extra-intestinal sites [48]. This study showed that microbial richness increased in the CB compared with the CON, indicating greater stability in the gut and ability to recover from infections. Research has shown that a reduction of diversity in the gut microbiota of patients with inflammatory bowel disease [40]. The microbial richness increase in the gut might account for greater stability in the digestive tract, which enhances the ability to recover from infectious postweaning diarrhea [46]. Previous research demonstrated that consumption of *C. butyricum* benefited the ecosystem of the intestinal tract by increasing the populations of probiotics and reducing those of unwanted bacteria [49]; Adding *C. butyricum* to feed of weaned piglets can increase the content of *Lactobacillus* [50], and also increase the diversity of intestinal bacteria [51]. *Lactobacillus casei* reduced the cytokine production in vitro for specimens of intestinal tissue from patients with ileal Crohn’s disease [52]. Direct-fed microbials that contain *C. butyricum* can reduce both severity and duration of diarrhea in children hospitalized with acute diarrhea, and increase fecal count of *Lactobacillus* by improvement in diarrheal disease [52]*. Bacillus* is one of the a member of direct-fed microbials [53], and the increase of *Bacillus* in the CB indicated the beneficial effect of *C. butyricum. Fusicatenibacter* and Ruminococcaceae are types of fermentative bacteria in the hindgut, which can help the host obtain more energy from complex polysaccharides resistant to the action of digestive enzymes [29, 54], and increased feed efficiency might be associated with increases in *Fusicatenibacter* and Ruminococcaceae. Research has shown that dysbiosis in rats decreased the level of Ruminococcaceae and increased intestinal permeability [55]. The increasing of Ruminococcaceae in the CB treatment might indicate that *C. butyricum* decreased the dysbiosis.

## Conclusions

Dietary supplementation with *C. butyricum* had positive effects on growth of weaned pigletswith less digestible diets. There was a tendency to reduce F/G, which could reduce feed costs in pig production. The beneficial effect may result from decreasing of post-weaning diarrhea by improving the intestinal morphology, intestinal microflora profile and immune function.

## Abbreviations

AB: Antibiotic treatment
ADFI: Average day feed intake
ADG: Average day gain
BW: Body weight
*C. butyricum*: *Clostridium butyricum*
CB: *C.butyricum* treatment
CB.C: CB with LPS-challenged
CD: Crypt depth
CON: Control treatment
CON.C: CON with LPS-challenged
F/G: Feed-gain ratio
IL-10: Interleukin-10
IL-6: Interleukin-6
LPS: Lipopolysaccharide
TLR2: Toll-like receptor 2
TLR4: Toll-like receptor 4
TNF-α: Tumor necrosis factor-α
VH: Villus height
VH/CD: Villus height/ crypt depth
